# Supplementary material for: Design Principles of Large Cation Incorporation in Halide Perovskites
Source: Molecules. 2021 Oct 13;26(20):6184. doi: 10.3390/molecules26206184 (PMC8539499; doi:10.3390/molecules26206184)
Supplement: Supplementary file 1 [file molecules-26-06184-s001.zip › molecules-1386568-supplementary.pdf]

Supplementary Information for

Design Principles of Large Cation Incorporation in Halide Perovskites

Heesoo Park, Syam Kumar R., Sanjay Chawla and Fedwa El-Mellouhi

Fedwa El Mellouhi
E-mail: felmellouhi@hbku.edu.qa

This PDF file includes:

Figs. S1 to S5
SI References

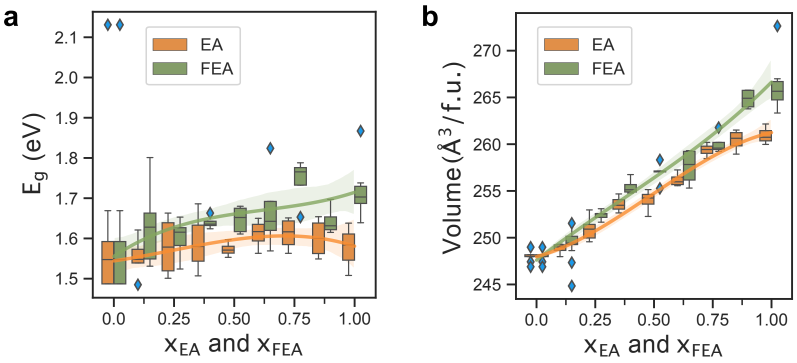

Fig. S1. (**a**) Bandgap E_g of $MA_{1-x_{EA}}EA_{x_{EA}}PbI_3$ and $MA_{1-x_{FEA}}FEA_{x_{FEA}}PbI_3$, where $0 \leq x_{EA} \leq 1$ and $0 \leq x_{FEA} \leq 1$, respective. (**b**) Comparison of unit cell volume increase.

A. Bandgap. As for any photoactive compound, its electronic properties determine the prospects of its domain of application. Figure S1**a** presents the calculated bandgaps of the EA- and FEA-substituted perovskites considered in this work. In general, the bandgaps get larger as x_{EA} and x_{FEA} increase. One can note that the bandgap change the EA-incorporated perovskites behaves in a bow shape reaching a maximum at $x_{EA} = 0.675$ then it decreases when $x_{EA} < 0.657$. On the other hand, E_g of the FEA-incorporated perovskites behaves differently from the EA-incorporated perovskites by showing a monotonous increase probably because of a severely distorted local structure and a buildup of strain. Figure S1**b** shows the size impact of $[EA]^+$ (274 pm) and $[FEA]^+$ (338 pm), as the unit cell volume increases. The bandgap reflects the perovskite's cation composition as $[PbI_3]^-$ network responds to the incorporated cations and its consequential octahedral distortion (1). The local atomistic structures are determined by the type of bonding (covalent, ionic, hydrogen, halogen-halogen), electrostatic potential, dipole moment, effective ionic radius. It is worth accordingly looking into the distortion of atomistic structures to understand the local strain by comparing the impact between EA and FEA to understand the intrinsic stability of perovskite and the bandgap change.

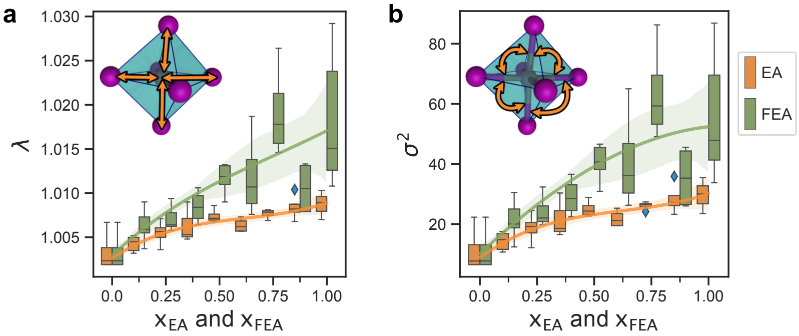

Fig. S2. Correlation of octahedral deformaton on the EA and FEA concentration in a perovskite. We identified the octahedral deformations: (**a**) quadratic elongation λ and (**b**) angle variance σ^2.

B. Structural deformation. To analyze the octahedral deformation, we identified the $[PbI_6]$ octahedra by using Voronoi analysis, and, subsequently, we analyzed the coordinates. Accordingly, we could calculate the octahedra metrics (2). The quadratic elongation λ is defined as:

$$\lambda = \frac{1}{6}\sum_{i=1}^{6}\left(\frac{l_i}{l_0}\right)^2 , \qquad [1]$$

where l_i is the distance between the central atom and i-th coordinated anion in an octahedron, and l_0 is the same distance for the ideal cubic octahedron with the same volume. Besides, the angle variance σ^2 is:

$$\sigma^2 = \frac{1}{11}\sum_{i=1}^{12}(\phi_i - 90)^2 , \qquad [2]$$

where ϕ_i is the i-th I−Pb−I angle. An ideally cubic perovskite should have a quadratic elongation $\lambda = 1$, an angle variance $\sigma^2 = 0$.

Figure S2 presents the changes of λ and σ^2 as a function of the additive cations' concentrations, by varying x_{EA} and x_{FEA} in $MA_{1-x_{EA}}EA_{x_{EA}}PbI_3$ and $MA_{1-x_{FEA}}FEA_{x_{FEA}}PbI_3$, respectively. Because λ and σ^2 values are observed correlate linearly with

each other, we observe very similar trends. Meanwhile, the impact of FEA on the perovskite is prominently distinguishable from that of EA.

For example, at low additive cation concentration, namely, $x_{\mathrm{EA}} = 0.125$ and $x_{\mathrm{FEA}} = 0.125$, the significant contrast emerges between the impact of EA and FEA, in both λ and σ^2. The extent of octahedral deformation is overlapped between $\mathrm{MAPbI_3}$ and $\mathrm{MA_{0.875}EA_{0.125}PbI_3}$. Likewise, the overlap runs over up to the high x_{EA} concentration as well. The successfully fabricated perovskites with a large amount of EA (3–5) corroborate this observation.

In the meantime, the FEA-incorporated perovskites are contrasting to the counterpart of EA-incorporated perovskites. The FEA alignments in a perovskite causes the instability of the perovskite. Although the λ and σ^2 values are relatively gathered in the range of $0 \leq x_{\mathrm{FEA}} \leq 0.375$, $\mathrm{MA_{0.875}FEA_{0.125}PbI_3}$ surpasses the interquartile range of $\mathrm{MAPbI_3}$. Moreover, the widely distributed values imply the impact of FEA alignments and its orientational glass formation as reported in hybrid organic-inorganic halide perovskite at the varying temperature (6). As we note that the FEA's alignment is less significant at low FEA concentration, less FEA incorporation would be affordable within a perovskite than the EA-incorporated counterpart perovskites. However, high concentration FEA stresses the $\mathrm{PbI_3}$ network strongly in the 3D perovskite framework.

The organic cations are interacting with the $\mathrm{PbI_3}$ network chain mainly by non-covalent bonds, of which formation is typically even weaker than Pb−I bond formation. And the impact of EA on a perovskite shows the strong correlation between the EA concentration and structural deformation induced by the cation, Thus, we suggest that the proportionate octahedral deformation can be used to indicate a cation's suitability in a perovskite framework. When $x_{\mathrm{FEA}} = 0.375$, the λ and σ^2 values approximate the values at $x_{\mathrm{EA}} = 1.0$ in the EA-incorporated perovskites. So, for example, if EA-incorporation is available up to $x_{\mathrm{EA}} = 0.2$, then, we suggest that FEA can be accommodated up to $x_{\mathrm{FEA}} = 3/8 * 0.2$ amount in $\mathrm{MA_{1-x}FEA_xPbI_3}$ perovskites. And, subsequently, exploiting the set of our DFT calculations, we propose the comparison of the octahedra deformation metrics, such as λ and σ^2, which can be used to explore the formable perovskites.

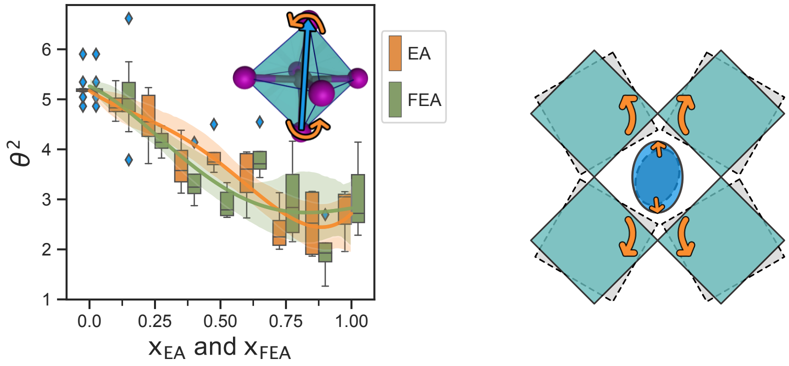

Fig. S3. Correlation of octahedral tilt θ^2 on the EA and FEA concentration in a perovskite. The cartoon sketches the mechanistic recovery of the octahedral tilt.

And we measured the octahedra tilt with respect to the lattice vectors as we define:

$$\theta^2 = \frac{1}{3}\sum_{i=1}^{3}\left[\arccos\left(\frac{\vec{v}_i \cdot \vec{a}_i}{|\vec{v}_i||\vec{a}_i|}\right)\right]^2 . \qquad [3]$$

The vector $\vec{a}_i$ is the lattice vector whose direction makes the smallest angle with a diagonal ($\vec{v}_i$) in the octahedron. An ideally cubic perovskite should have an octahedral tilt $\theta^2 = 0$.

We can relate this θ^2 value to the tolerance factor (τ). An ideal cubic perovskite would have $\tau \approx 1$. A perovskite with smaller τ values likely belongs to lower symmetry structures with the tilted $\mathrm{PbI_6}$ octahedra. Contrarily, a material with larger τ values would turn into hexagonal phase. Alongside the tolerance factor, we can take the effective τ of the mixed-cation perovskite by defining the average cation radius of the mixture as the composition-weighted average of the individual radii for $\mathrm{MA}_{1-x}A_x$ as:

$$r_{\mathrm{MA}_{1-x}A_x} = (1-x)r_{\mathrm{MA}} + x r_A, \qquad [4]$$

where $0 \leq x \leq 1$, and r_i is the effective ionic radius of i-composite cation. According to this definition, τ becomes 1 when $x_{\mathrm{EA}} = 0.906$ and $x_{\mathrm{FEA}} = 0.641$. Figure S3 displays the characterized tilted cubic structure subgroups with these concentrations.

Interestingly, the concentration of FEA ($x_{\mathrm{FEA}} = 0.641$) agrees to the point where the θ^2 behavior changes abruptly. The θ^2 values decrease monotonously in the range $0 \leq x_{\mathrm{FEA}} \leq 0.5$. As we observed the face-alignment distribution, these cations align toward with faces of the cube by avoiding the short-range repulsion between cation and $\mathrm{I^-}$. At the same time, the cation stretches the tilted octahedra, as the cartoon in Figure S3 sketches the mechanistic recovery of the octahedral tilt by EA and FEA.

In contrast, in the range $0.5 < x_{\mathrm{FEA}} \leq 1.0$, the values change is no longer monotonous while the broad distribution implies the significance of the cation alignments in the $\mathrm{PbI_3}$ network. Meanwhile, we note that the monotonous decrease of θ^2 disappears when $0.875 < x_{\mathrm{FEA}} \leq 1.0$ because $\tau = 1$ when $x_{\mathrm{EA}} = 0.906$ in the EA-incorporated perovskites. At these high

concentrations, the perovskites encounter the lattice strain stress turning into a hexagonal phase induced by the cations' sizes and their alignments. Hence, the cusp in θ^2 reveals the perovskite structure remains stable no longer. While we focus on the large cations, we could find this inclination where octahedra tilt values are widely scattered by the cation's alignment order at the high concentration. In addition to λ and σ^2, this θ^2 is an essential indicator of cation suitability.

C. Non-covalent interactions. To understand the observation of the FEA-incorporated compounds in the mixing enthalpy and octahedral deformation, we carried out the non-covalent interaction (NCI) analysis (7, 8). The NCI method maps the local bonding properties by reduced-density gradient (RDG, s) from electron density (ρ), defined as:

$$s = \frac{1}{2(3\pi)^{1/3}} \frac{|\nabla \rho|}{\rho^{4/3}}. \qquad [5]$$

This combination of s and ρ partitions non-covalent interactions (low-s and low-ρ) from covalent interactions (low-s and high-ρ).

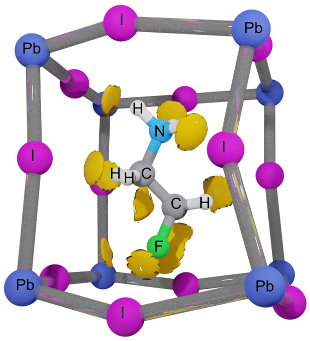

Fig. S4. NCI isosurfaces ($s = 0.6$) around the FEA in $MA_{0.875}FEA_{0.125}PbI_3$. Only the isosurface around the FEA cation is illustrated in yellow for clarity.

We have seen a high FEA-concentration induces the structural deformation. Hence, to avoid the influence of the steric effect of an FEA's high concentration while we scrutinize the fluorine's non-covalent interactions, the NCI analysis is focused on the $MA_{0.875}FEA_{0.125}PbI_3$ compound at the ground state.

As shown in Figure S4, we mapped the NCI isosurface (yellow) of $MA_{0.875}FEA_{0.125}PbI_3$ at the ground-state structure to visualize the halogen-halogen bonding as well as hydrogen bonding. The NCI domains of F···I contacts are essentially similar to the hydrogen bonding, showing the strong directional attractions.

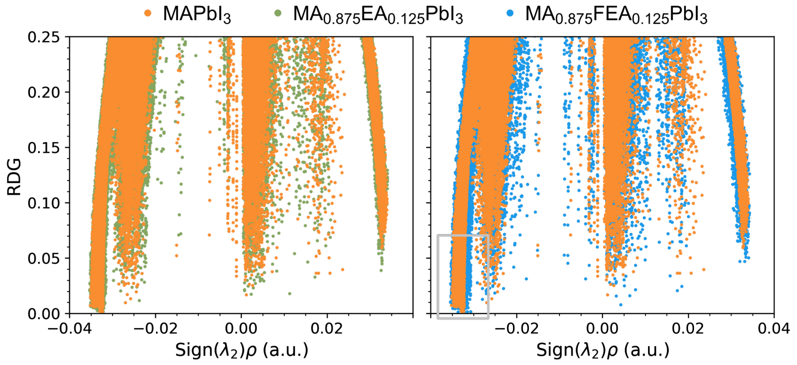

Fig. S5. Enhanced non-covalent interactions with FEA. Reduced density gradient plots as a function of $\mathrm{Sign}(\lambda_2)\rho$ are compared in the range $-0.04 < \rho < 0.04$. $MAPbI_3$, $MA_{0.875}EA_{0.125}PbI_3$, and $MA_{0.875}FEA_{0.125}PbI_3$ compounds are marked in orange, green, and blue, respectively. The gray box denotes the broaden range of non-covalent interactions in $MA_{0.875}FEA_{0.125}PbI_3$.

And, in Figure S5, s is multiplied by the sign of the second density Hessian eigenvalue (λ_2) at each grid point. We compare between $MAPbI_3$ and $MA_{0.875}EA_{0.125}PbI_3$, and between $MAPbI_3$ and $MA_{0.875}FEA_{0.125}PbI_3$. Therefore, this NCI analysis reveals the impact of EA and FEA on the hydrogen bonding and halogen bonding in the ground-state perovskites quantitatively, as the interactions are featured at $\mathrm{Sign}(\lambda_2)\rho \approx -0.03$. In addition, around at $\mathrm{Sign}(\lambda_2)\rho = 0$, the peaks describes the repulsive (ρ^+) and attractive (ρ^-) weak-interactions.

While both EA and FEA appear their steric repulsion over MA's repulsive interactions in the inorganic network, we observe the repulsive interactions are limited only at the weak non-covalent interactions. In contrast, it is notable that the FEA cation broadens the peak of the attractive interactions. We highlight it by the gray box in the comparison plot of $MAPbI_3$ and $MA_{0.875}FEA_{0.125}PbI_3$. This broad strength of attractive interaction represents the halogen-halogen bond in F···I. Hence, an FEA cation still sustains in the PbI_3 perovskites' network.

References

1. H Park, et al., Importance of structural deformation features in the prediction of hybrid perovskite bandgaps. *Comput. Mater. Sci.* **184**, 109858 (2020).
2. K Robinson, GV Gibbs, PH Ribbe, Quadratic elongation: A quantitative measure of distortion in coordination polyhedra. *Science* **172**, 567–570 (1971).
3. Z Chu, et al., Large cation ethylammonium incorporated perovskite for efficient and spectra stable blue light-emitting diodes. *Nat. Commun.* **11**, 4165 (2020).
4. S Xu, et al., Ea-directing formamidinium-based perovskite microwires with a-site doping. *ACS Omega* **6**, 7157–7164 (2021).
5. K Nishi, T Oku, T Kishimoto, N Ueoka, A Suzuki, Photovoltaic characteristics of ch3nh3pbi3 perovskite solar cells added with ethylammonium bromide and formamidinium iodide. *Coatings* **10** (2020).
6. EM Mozur, et al., Orientational Glass Formation in Substituted Hybrid Perovskites. *Chem. Mater.* **29**, 10168–10177 (2017).
7. ER Johnson, et al., Revealing noncovalent interactions. *J. Am. Chem. Soc.* **132**, 6498–6506 (2010).
8. A Otero-de-la Roza, ER Johnson, J Contreras-García, Revealing non-covalent interactions in solids: Nci plots revisited. *Phys. Chem. Chem. Phys.* **14**, 12165–12172 (2012).
